# Pathophysiological mechanisms of post-myocardial infarction depression: a narrative review

**DOI:** 10.3389/fpsyt.2023.1225794

**Published:** 2023-08-04

**Authors:** Eric Garrels, Tejasvi Kainth, Briana Silva, Garima Yadav, Gurtej Gill, Mona Salehi, Sasidhar Gunturu

**Affiliations:** ^1^Department of Psychiatry, BronxCare Health System, New York, NY, United States; ^2^BronxCare Health System, New York, NY, United States; ^3^Department of Psychiatry, Icahn School of Medicine at Mount Sinai, New York, NY, United States

**Keywords:** major depressive disorder, post myocardial infarction, HPA axis, coagulation, inflammation, pathophysiological

## Abstract

Myocardial infarction (MI) can have significant physical and mental consequences. Depression is a prevalent psychiatric condition after MI which can reduce the quality of life and increase the mortality rates of patients. However, the connection between MI and depression has remained under-appreciated. This review examines the potential connection between depression and MI by overviewing the possible pathophysiologic mechanisms including dysregulation of the hypothalamic-pituitary-adrenal axis and autonomic nervous system, coagulation system dysfunction, inflammation, environmental factors, as well as, genetic factors. Furthermore, depression can be an adverse event of medications used for MI treatment including beta-blockers, statins, or anti-platelet agents. The need for early detection and management of depression in patients with MI is, therefore, crucial for improving their overall prognosis. Adherence to treatments and regular follow-up visits can ensure the best response to treatment.

## 1. Introduction

Depression is a highly prevalent mental disorder that imposes significant economic and social burdens (1). Over 300 million (4.4%) individuals are estimated to be affected by depression worldwide, with higher rates among females (5.1%) than males (3.6%). Additionally, the prevalence of depressive disorders increases with age, affecting over 7.5% of females and 5.5% of males aged over 55 years (2). According to projections, depressive disorders are expected to become the first leading cause of the burden of disease in high-income countries by 2030 and the second leading cause worldwide (3).

Cardiovascular disorders are the leading causes of mortality worldwide (4). Ischemic heart disease (IHD) is a subtype of cardiovascular disorder and was found to cause about 8.4 to 9.7 million deaths in 2019 and was more common in developed countries than in developing nations (4). Myocardial infarction (MI) as the most severe form of IHD can cause several physical and mental issues. Depression is one of the most prevalent psychological reactions after MI (5–10). A recent meta-analysis of over 12,000 subjects with MI reported that about 29% of individuals experienced depression (11). Depression after MI can lead to lower quality of life and increase the mortality of patients (8–10). Post-MI depression was found to be associated with a 2 to 2.5-fold increased risk of cardiovascular complications (12).

Despite its prevalence, post-MI depression is often overlooked as a natural emotional reaction to physical illness. Furthermore, emerging evidence has shown that depression is a risk factor for MI (13). In this study, we aim to shed light on the pathways connecting MI and depression by outlining the possible pathophysiologic mechanisms. Our study provides an updated comprehensive overview of the association between post-MI depression and different factors, including dysregulation of the hypothalamic-pituitary-adrenal axis and autonomic nervous system, coagulation system dysfunction, inflammation, as well as, genetic factors (Table 1).

**Table 1:** Neurobiological mechanisms of post-MI depression.

**Role of the autonomic nervous system and HPA axis**	Continuous upregulation of the HPA and SA systems Activation of pro-inflammatory cytokines and the HPA and autonomic nervous systems Emotional stress and serotonin dysregulation in depression Hypercortisolemia	**Role of coagulation system**	↓ levels of BDNF and tPA in emotional stress and depression ↑ levels of PAI-1 in depression ↑ levels of PAI-1 lead to ↓ levels of BDNF	**Role of inflammation**	Lack of oxygen—DAMPS release—immune cells activation—pro-inflammatory cytokines—↑ release of IL-1, IL-6, TNF-α, CRP—↑ kynurenine pathway activation—↑ NMDA receptor agonists production Pro-inflammatory cytokines—altered neurotransmitters metabolism and production Pro-inflammatory cytokines—↓ levels of BDNF	**Genetic correlation**	Depression linked with ↑ risk of heart failure and small vessel stroke Depression and CVD—↑ levels of IL-6, CRP, and triglycerides Variant serotonin transporter gene (5-HTTLPR) ↑ risk of depression after MI, poor response to antidepressants Variant serotonin receptor gene (5-HT2A)—↑ risk of depression after MI IL-1 gene—↑ risk of depression after MI, pro-inflammatory response

HPA, hypothalamic-pituitary-adrenal; SA, sympathetic-adrenomedullary; TNF-α: tumor necrosis factor-alpha, interleukin 6 (IL-6), interleukin 1 (IL-1), C-reactive protein (CRP), LDL, low-density lipoprotein; HDL, high-density lipoprotein; PVCs, premature ventricular contractions; GP IIb/IIIa, glycoprotein IIb/IIIa; GP Ib/IX, glycoprotein Ib/IX; BDNF, brain-derived neurotrophic factor; tPA, tissue-type plasminogen activator; PAI-1, plasminogen activator inhibitor-1; DAMPs, damage-associated molecular patterns; MI, myocardial infarction; CVD, cardiovascular disorders.

## 2. Hypothalamic-pituitary-adrenal axis and autonomic nervous system

**Figure 1:**
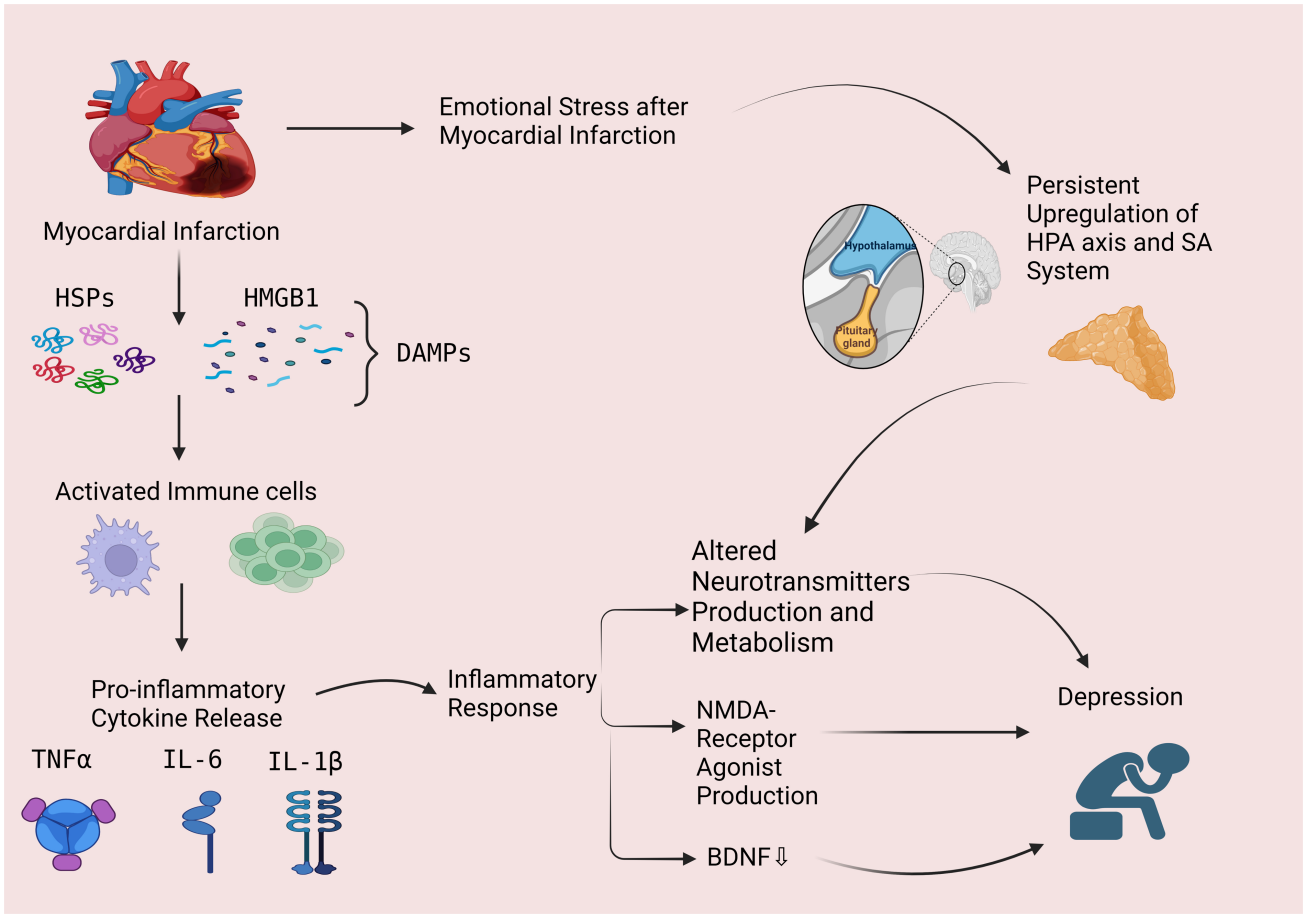
The possible role of inflammatory response and hypothalamic pituitary axis as possible mechanisms in post-MI depression. Myocardial infarction leads to a lack of oxygen and the release of damage-associated molecular patterns (DAMPs), such as high-mobility group box 1 (HMGB1) and heat shock proteins (HSPs). These DAMPs activate immune cells, such as macrophages, which phagocytose the damaged tissue and release pro-inflammatory cytokines, such as interleukin-1β (IL-1β), interleukin-6 (IL-6), and tumor necrosis factor-α (TNF-α), leading to an inflammatory response. Inflammation can lead to depression through 3 mechanisms including: (1) altering the production and metabolism of neurotransmitters, (2) increased production of N-methyl-D-aspartate (NMDA) receptor agonist and, (3) decreased levels of brain-derived neurotrophic factor (BDNF). Myocardial Infarction also cause emotional stress which activates the hypothalamic-pituitary-adrenal (HPA) axis and sympathetic-adrenomedullary (SA) system and can cause altered metabolism and production of neurotransmitters, leading to depression. HPA, hypothalamic-pituitary-adrenal; SA, sympathetic-adrenomedullary; DAMPs, damage-associated molecular patterns; HMGB1, high-mobility group box-1; HSPs, heat shock proteins, TNF-α, tumor necrosis factor-alpha, interleukin 6 (IL-6), Interleukin 1β (IL-1β), NMDA, N-methyl-D-aspartate; BDNF, brain-derived neurotrophic factor. (This figure is created by Bioreneder.com).

MI elicits various consequential responses, such as the activation of the hypothalamic-pituitary-adrenal (HPA) axis and dysregulation of the autonomic nervous system (ANS) (14). These effects can further cause selective dysfunction in the prefrontal cortex and anterior cingulate gyrus which develop depression (14–16) (Figure 1).

According to a recent study on post-MI patients (15), an immediate increase in cortisol concentration due to the activation of the HPA axis was observed after MI, which returned to baseline within 72 h. There was no difference between morning and afternoon cortisol levels of individuals with post-MI depression. Patients with depression lasting for more than 3 months exhibit a more pronounced flattened daily rhythm of cortisol secretion. Among post-MI patients without depression, however, the afternoon cortisol level was significantly lower than the morning level. An abnormal cortisol rhythm has been linked to cognitive impairment and reduced stress-coping abilities, which may increase the risk of developing depressive symptoms (15, 17).

Emotional stress associated with post-MI depression can activate the sympathetic-adrenomedullary (SA) system, which in conjunction with the HPA axis activation, can cause dysregulation of serotonin and may contribute to the maintenance of depressive symptoms (18–20).

## 3. Environmental factors

Several lifestyle and environmental factors have been identified as potential contributors to the development of post-MI depression, including lack of social support, lifestyle changes, financial stress, and health-related fears and anxiety (21–29).

### 3.1. Lack of social support

Lack of social support or a poor support system has been linked to an increased risk of post-MI depression (21). Limited emotional or practical support from family, friends, or healthcare providers can exacerbate feelings of isolation, sadness, and distress. On the other hand, strong social support can help individuals cope better with the emotional challenges following MI (21, 22).

### 3.2. Lifestyle changes

Following MI, individuals are often advised to make significant lifestyle modifications, such as adopting a healthier diet, engaging in regular physical activity, quitting smoking, and reducing alcohol consumption (23). Difficulties in implementing and maintaining these lifestyle changes and unhealthy lifestyle habits, including physical inactivity, poor dietary choices, smoking, and excessive alcohol consumption, have been associated with a higher risk of post-MI depression. These habits can worsen physical health outcomes, impact mood regulation, and contribute to a negative emotional state (23, 24).

### 3.3. Financial stress

Financial strain resulting from medical expenses, loss of income, or inability to work due to a heart attack can contribute to post-MI depression (25). Financial difficulties can heighten anxiety, worry, and uncertainty about the future, which may negatively impact mental well-being (25).

### 3.4. Health-related anxiety

After experiencing MI, individuals may develop health-related anxiety, including fear of another cardiac event, fear of physical exertion, or hypochondriasis. These fears can lead to increased distress, avoidance of physical activity, and impaired quality of life, potentially contributing to the development of depression (26).

Additionally, several other factors can exacerbate these environmental factors and influence the development of post-MI depression. Pre-existing mental health conditions, particularly a prior history of anxiety or depression (27, 28), as well as complications during hospitalization (29), are notable examples.

## 4. Coagulation system

Tissue-type plasminogen activator, or tPA, is a thrombolytic enzyme that converts plasminogen to plasmin and plays an important role in promoting neuronal synaptic plasticity (30). The plasminogen activator inhibitor 1 (PAI-1) is a major endogenous inhibitor of tPA within the extracellular space (31) and is encoded by the *SERPINE1* gene. *SERPINE1* has been linked to increased susceptibility to depression and may influence the therapeutic response to SSRIs (30, 31). While the relationship between tPA levels and depression remains unclear, evidence suggests that PAI-1 levels increase during psychological stress and depression (30). Lower PAI-1 levels in patients with anxiety and depression who were treated with serotonergic antidepressants have been reported (32). One study found that depressed patients had lower tPA levels prior to antidepressant treatment. After 8 weeks of treatment, however, levels of tPA significantly increased (33). This may show the possible correlation between depression and MI, as high fibrinogen levels and high PAI-1 levels present an increased risk for ischemic cardiovascular events such as MI. Because PAI-1 inhibits tPA, there is an important link between the fibrinolytic processes of this inhibition and the increased risk for cardiovascular disease.

The possible correlation between the coagulation system and the development of depression can be further discovered via the production of brain-derived neurotrophic factor (BDNF), as the tPA-plasmin pathway cleaves the precursor to BDNF, pro-BDNF, to BDNF (34, 35). Neurotrophins are key regulators of synaptic plasticity and neuronal connectivity (34), and BDNF is a small dimeric neurotrophin that is strongly implicated in the pathophysiology of depression due to its high expression in brain regions responsible for mood regulation, including the hippocampus, prefrontal cortex, and amygdala (35, 36). Preclinical and clinical studies have consistently shown that levels of BDNF decrease within the brain during periods of emotional and psychological stress and depression (34–36).

## 5. Inflammation

Inflammation plays a crucial role in the pathophysiology of MI and subsequent depression (37). During MI, the lack of oxygen and nutrients causes damage to the heart muscle, leading to the release of damage-associated molecular patterns (DAMPs), such as high-mobility group box 1 (HMGB1) and heat shock proteins (HSPs) (37). These DAMPs activate immune cells, such as macrophages, which phagocytose the damaged tissue and release pro-inflammatory cytokines, such as interleukin-1β (IL-1β), interleukin-6 (IL-6), and tumor necrosis factor-α (TNF-α) (37, 38) (Figure 1).

Inflammatory cytokines have been shown to contribute to the development of depression in both animal and human studies. Studies in rodents have demonstrated that administration of IL-1β or TNF-α induces depressive-like behavior, while blockade of these cytokines attenuates depressive-like behavior in response to stress (39, 40). In humans, elevated levels of inflammatory cytokines have been found in patients with depression, including those with post-MI depression (41). The mechanisms by which inflammatory cytokines contribute to depression are complex and not fully understood. It is thought that these cytokines may activate the kynurenine pathway, leading to increased production of quinolinic acid, an N-methyl-D-aspartate (NMDA) receptor agonist that has been implicated in the pathophysiology of depression (42). In addition, inflammatory cytokines can also affect the production and metabolism of neurotransmitters, such as serotonin, dopamine, and norepinephrine, which are involved in the regulation of mood (43, 44). For example, IL-6 can induce the expression of indoleamine 2,3-dioxygenase (IDO), an enzyme that metabolizes tryptophan, the precursor of serotonin. This can lead to a decrease in serotonin production and an increase in the production of kynurenine, which has been linked to the development of depression (45). Furthermore, inflammatory cytokines have been linked to decreased levels of brain-derived neurotrophic factor (BDNF), a protein that is important for the survival and function of neurons. Studies have shown that decreased levels of BDNF are associated with depression, and inflammatory cytokines can decrease the expression of BDNF in the brain (46–48).

In summary, inflammation and inflammatory cytokines play a crucial role in the development of post-MI depression. Future research on the relationship between inflammation and post-MI depression may lead to new treatments and preventative strategies for this debilitating condition.

## 6. Genetic factors

Recent studies utilizing Mendelian randomization (MR) have discovered a noteworthy correlation between genetic susceptibility to depression and an increased risk of cardiovascular disorders (CVD) and MI. Moreover, genetic-related depression is linked to a higher risk of heart failure and small-vessel stroke. These findings demonstrated that depression has enduring and stable effects on the risk of MI (49, 50).

Genetic and environmental factors contribute to the pathophysiology of depression after MI. One study found that a variant of the serotonin transporter gene or *5-HTTLPR* was associated with an increased risk of depression after MI and that patients with this variant had a poorer response to antidepressant treatment (51). Another study found that genetic variations in the interleukin-1 (IL-1) gene were associated with an increased risk of depression after MI, possibly due to the role of IL-1 in the inflammatory response (52). A study found that patients with a family history of depression were more likely to develop depression after MI and this risk was further increased in patients who experienced a high level of stress during the MI (53). Based on a review by Schins et al. (54), the increased risk of thromboembolic events in patients with depression and cardiovascular disease may be linked to the upregulation and/or heightened sensitivity of serotonin receptors 5-HT2A/1B, as well as the downregulation of serotonin transporter (5-HTT) receptors. Additionally, the S allele of the serotonin transporter (5-HTT) gene-linked polymorphic region was found to be associated with both depressive symptoms and cardiac events (55). Although these studies suggest a role for genetics in the pathophysiology of depression after MI, it is important to note that depression is a complex disorder and further research is needed to fully understand the genetic basis of post-MI depression and to identify potential targets for treatment and prevention.

## 7. Post-MI medications and their potential association with post-MI depression

Certain medications prescribed after MI may contribute to the development of depression in patients. The use of beta-blockers after MI is a standard therapeutic approach aimed at reducing the risk of future cardiovascular events and improving overall cardiac function (56). Research examining the relationship between beta-blocker use and depression after MI has produced mixed results. Some studies have reported a higher incidence of depressive symptoms in patients treated with beta-blockers (57–59) while others have found no significant association (60–64). It is important to note that the evidence is not conclusive and further research is needed to establish a clearer understanding of this relationship. The mechanism behind the potential association between beta blockers and depression is not fully understood. It has been proposed that beta-blockers may have an impact on the central nervous system, influencing neurotransmitters and hormonal pathways that are involved in mood regulation. However, the exact biological mechanisms linking beta-blocker use and depression after MI remain speculative and require more investigation.

Statins are other commonly prescribed medications that are used to decrease cholesterol levels and prevent cardiovascular events, including MI (65). Several studies have investigated the potential link between statin use and the risk of depression (66–74). The findings have been controversial, with some studies suggesting a possible protective effect of statins against depression (66–70) while others have found no significant association (70) or even an increased risk (71–74). One proposed mechanism by which statins might influence depression risk is through their anti-inflammatory properties. It is believed that inflammation plays a role in the development of depression, and statins have been shown to reduce the expression of hippocampal pro-inflammatory cytokines such as IL-1β, TNF-α, and IL-6. By modulating the inflammatory response, statins could potentially have a positive impact on mood and depressive symptoms (67). Given the conflicting findings in the existing literature, more research is needed to better understand the relationship between statin use after MI and the risk of depression.

Antiplatelet agents, such as aspirin, are often used after MI to prevent blood clots and lower the risk of recurrent MI (75). Aspirin was found to protect against depression based on several studies (67, 74), while others found no effect (76, 77) or even an increased risk of depression (78). One of the possible mechanisms for the depressogenic effects of aspirin can be its potential impact on the arachidonic acid pathway. Arachidonic acid, which is associated with mood disorders, has been linked to depression when its levels are higher compared to other fatty acids (79). Moreover, arachidonic acid can directly affect brain serotonin transporters (80). Therefore, by inhibiting the metabolism of arachidonic acid, aspirin could potentially interfere with serotonin systems that regulate mood (80).

## 8. Implications for clinical practice

An overview of clinical points can be seen in Table 2. Post-MI depression is common and associated with poor outcomes and increased healthcare costs. Ongoing research aims to improve clinical practice and patient outcomes, with some implications including:

**Table 2:** Clinical summary of the relationship between depression and myocardial infarction.

Clinical summary
**Understanding the link between depression and myocardial infarction:**	Many preclinical and clinical studies have demonstrated a strong, bidirectional link between major depressive disorder and coronary artery disease. Depression is a prevalent mental illness and a leading cause of disability that affects the global population. Furthermore, depression serves as both an independent risk factor for cardiovascular disease and a worsening prognostic factor that increases morbidity and mortality in patients already diagnosed with cardiovascular disease. Despite recognizing the well-established two-way relationship and global health concern between depression and cardiovascular disease, the exact mechanisms of action require more definitive exploration and explanation. The bidirectional, complex interplay that exists between major depressive disorder and myocardial infarction can be outlined by several key mechanisms, including, Environmental factors Inflammation Persistent overregulation of autonomic nervous systems and HPA axis Increased platelet aggregation and thrombosis The tissue-type plasminogen activator (tPA), and plasminogen activator inhibitor-1 (PAI-1) MI-related medications Fully optimized doses of SSRIs with cognitive therapy serve as an effective combinational treatment for depression in cardiac patients.

1. Screening: Detecting depression early is vital for prompt treatment. Tools like PHQ-9 and HADS can screen and identify patients with depression (81, 82).
2. Treatment: Post-MI depression treatment involves pharmacological and non-pharmacological interventions, such as selective serotonin reuptake inhibitors (SSRIs) and serotonin and norepinephrine reuptake inhibitors (SNRIs). Effective psychosocial interventions include cognitive behavioral therapy (CBT) and cardiac rehabilitation programs (83).
3. Adherence: Adherence to treatment is crucial for the successful management of depression. Patients should be educated about the importance of adherence to their medication and therapy sessions (84).
4. Follow-up: Regular follow-up visits with healthcare providers is essential for the monitoring of depression symptoms and the adjustment of treatment as needed (84).

Tailored treatment approaches based on individual needs could enhance post-MI depression treatment outcomes. Identifying biomarkers to match specific treatments is actively investigated. Digital interventions like mHealth apps and telehealth can boost treatment access and adherence by providing real-time support and feedback (85–87). Combining pharmacological and non-pharmacological approaches can improve outcomes. For instance, combining CBT with antidepressants has proved effective in post-MI depression treatment (83). Therefore, prompt identification and treatment of post-MI depression are crucial, and personalized, digital, and combined interventions can improve outcomes (83). Further research, including RCTs, will provide a better understanding of the best post-MI depression treatment approaches.

## 9. Discussion

The precise cause of post-MI depression remains unclear. Other possible underlying mechanisms have been proposed in some studies such as the possible role of abnormal lipid profiles in developing depression (88–90). Some studies have reported a positive correlation between unipolar depression and elevated levels of low-density lipoprotein (LDL) cholesterol (88–90) and total cholesterol (91), along with lower levels of high-density lipoprotein cholesterol (HDL) (86, 87). However, some evidence reported a negative correlation between LDL (92, 93) and total cholesterol (94–98) levels with depression and suicidal behaviors. There is also an increasing amount of literature suggesting no correlation between serum lipids and depressive episodes (99–104). These discrepancies in the findings could be attributed to various factors, including uncontrolled confounding variables and differences in study settings. To gain a better understanding of the relationship between lipid profiles and depression, it is imperative to conduct further studies with larger sample sizes.

It should be noted that depression is also a risk factor for MI development (13). Several mechanisms including platelets activation and thrombosis, behavioral and lifestyle factors, inflammatory processes, as well as HPA axis and ANS have been proposed (105).

### 9.1. Platelets activation and thrombosis

There is a strong link between depression and platelet reactivity that can cause cardiovascular morbidities (106–109). Depressed patients have been found to demonstrate enhanced platelet reactivity and increased expression of activated glycoprotein (GP) IIb/IIIa, GP Ib/IX receptors, P selectin, β thromboglobulin and platelet factor four, and monoamine oxidase in comparison to healthy individuals (110, 111). GP Ib/IX receptors lead to a conformational change and activation of GP IIb/IIIa receptors. GP IIb/IIIa complex is a receptor for fibrinogen, fibronectin, vitronectin, Von Willebrand factor, and thrombospondin which enhance platelet activation (106, 108, 109). This heightened platelet activation may contribute to ischemic heart disease and post-MI mortality (112).

### 9.2. Behavioral and lifestyle factors

Chronic depression often leads to unhealthy lifestyle practices (113) such as physical inactivity (114), poor dietary habits, smoking, and non-adherence to medication regimens (115). These behaviors can increase the risk of developing cardiovascular disease, including MI (113). Additionally, depressed patients who have experienced an acute MI are less inclined to follow the suggested behavioral and lifestyle modifications aimed at decreasing the likelihood of future cardiac events (116).

### 9.3. Inflammatory processes

Chronic depression has been associated with an increased risk of systemic inflammation (117). Persistent elevation of pro-inflammatory markers, such as C-reactive protein (CRP), IL-6, and IL-1 in depression (118) can promote the development and progression of atherosclerosis, a condition characterized by the buildup of fatty plaques in the arterial walls. These plaques can eventually rupture, leading to the formation of blood clots that can block coronary arteries, resulting in myocardial infarction (118–120).

### 9.4. HPA axis and ANS

Previous studies have shown that patients with depression have an excessive rate of norepinephrine entry into plasma from the sympathetic nerves and a rapid elimination phase from the bloodstream which corresponds with an increased neuronal uptake (120). This excessive sympathetic outflow results in coronary vasoconstriction and reduced cardiac blood flow (121), major ventricular arrhythmias (121, 122), left ventricular hypertrophy (123), endothelial dysfunction (124), and MI (125). Current evidence also reported that changes in ANS can increase the risk of developing recurrent MI and higher mortality rate in post-MI patients (126–129). Individuals with depression often experience a reduction in cardiac parasympathetic tone and vagal activity leading to a decrease in heart rate variability (126–128). Decreased heart rate variability is strongly correlated with mortality in post-MI patients (126, 129). Furthermore, overstimulation of the HPA axis and increased levels of cortisol in depression (130, 131) can lead to metabolic syndrome (132). Metabolic syndrome in turn is associated with coronary heart disease (CHD), CVD, increased mortality rate (133) and increased sympathetic nervous system activity (134).

The relationship between chronic depression and MI is complex, and the mechanisms described above are not mutually exclusive. They likely interact and influence each other, contributing to an increased risk of MI in individuals with chronic depression (105). Additionally, other factors such as genetic predisposition (135), cardiovascular side effects of depression treatment (136), and comorbid conditions may also play a role in this association.

## Conclusion

Several factors including the dysregulation of the autonomic nervous system and HPA axis, inflammatory cytokines, coagulation system, platelet aggregation, various environmental factors, medications, and genetics can be contributed to the correlation between MI and depression. The co-occurrence of these two conditions can significantly impact the quality of life of the affected individuals.

## Author contributions

EG, TK, BS, GY, and GG: conceived and designed the study, collected and analyzed the data, and drafted the manuscript. MS and SG: contributed to the study design, editing, and critically revised the manuscript for important intellectual content and provided critical feedback on the manuscript and approved the final version for submission. All authors contributed to the article and approved the submitted version.

## Conflict of interest

The authors declare that the research was conducted in the absence of any commercial or financial relationships that could be construed as a potential conflict of interest.

## Publisher’s note

All claims expressed in this article are solely those of the authors and do not necessarily represent those of their affiliated organizations, or those of the publisher, the editors and the reviewers. Any product that may be evaluated in this article, or claim that may be made by its manufacturer, is not guaranteed or endorsed by the publisher.
